# Automated 4D flow cardiac MRI pipeline to derive peak mitral inflow diastolic velocities using short-axis cine stack: two centre validation study against echocardiographic pulse-wave doppler

**DOI:** 10.1186/s12872-023-03052-x

**Published:** 2023-01-16

**Authors:** Hosamadin Assadi, Rui Li, Ciaran Grafton-Clarke, Bhalraam Uthayachandran, Samer Alabed, Ahmed Maiter, Gareth Archer, Peter P. Swoboda, Chris Sawh, Alisdair Ryding, Faye Nelthorpe, Bahman Kasmai, Fabrizio Ricci, Rob J. van der Geest, Marcus Flather, Vassilios S. Vassiliou, Andrew J. Swift, Pankaj Garg

**Affiliations:** 1 Norwich Medical School, University of East Anglia, Norwich Research Park, Norwich, NR4 7UQ UK; 2 Norfolk and Norwich University Hospitals NHS Foundation Trust, Norfolk, UK; 3 Department of Infection, Immunity and Cardiovascular Disease, University of Sheffield Medical School and Sheffield Teaching Hospitals NHS Trust, Sheffield, UK; 4 Department of Clinical Radiology, Sheffield Teaching Hospitals NHS Foundation Trust, Sheffield, UK; 5 Leeds Institute of Cardiovascular and Metabolic Medicine, University of Leeds, Leeds, UK; 6 Department of Neuroscience, Imaging and Clinical Sciences, “G.d’Annunzio” University of Chieti-Pescara, Chieti, Italy; 7 Department of Radiology, Division of Image Processing, Leiden University Medical Center, Leiden, The Netherlands

**Keywords:** 4D flow CMR, Artificial intelligence, Peak velocity, Mitral valve, Doppler echocardiography

## Abstract

**Background:**

Measurement of peak velocities is important in the evaluation of heart failure. This study compared the performance of automated 4D flow cardiac MRI (CMR) with traditional transthoracic Doppler echocardiography (TTE) for the measurement of mitral inflow peak diastolic velocities.

**Methods:**

Patients with Doppler echocardiography and 4D flow cardiac magnetic resonance data were included retrospectively. An established automated technique was used to segment the left ventricular transvalvular flow using short-axis cine stack of images. Peak mitral E-wave and peak mitral A-wave velocities were automatically derived using in-plane velocity maps of transvalvular flow. Additionally, we checked the agreement between peak mitral E-wave velocity derived by 4D flow CMR and Doppler echocardiography in patients with sinus rhythm and atrial fibrillation (AF) separately.

**Results:**

Forty-eight patients were included (median age 69 years, IQR 63 to 76; 46% female). Data were split into three groups according to heart rhythm. The median peak E-wave mitral inflow velocity by automated 4D flow CMR was comparable with Doppler echocardiography in all patients (0.90 ± 0.43 m/s vs 0.94 ± 0.48 m/s, *P* = 0.132), sinus rhythm-only group (0.88 ± 0.35 m/s vs 0.86 ± 0.38 m/s, *P* = 0.54) and in AF-only group (1.33 ± 0.56 m/s vs 1.18 ± 0.47 m/s, *P* = 0.06). Peak A-wave mitral inflow velocity results had no significant difference between Doppler TTE and automated 4D flow CMR (0.81 ± 0.44 m/s vs 0.81 ± 0.53 m/s, *P* = 0.09) in all patients and sinus rhythm-only groups. Automated 4D flow CMR showed a significant correlation with TTE for measurement of peak E-wave in all patients group (r = 0.73, *P* < 0.001) and peak A-wave velocities (r = 0.88, *P* < 0.001). Moreover, there was a significant correlation between automated 4D flow CMR and TTE for peak-E wave velocity in sinus rhythm-only patients (r = 0.68, *P* < 0.001) and AF-only patients (r = 0.81, *P* = 0.014). Excellent intra-and inter-observer variability was demonstrated for both parameters.

**Conclusion:**

Automated dynamic peak mitral inflow diastolic velocity tracing using 4D flow CMR is comparable to Doppler echocardiography and has excellent repeatability for clinical use. However, 4D flow CMR can potentially underestimate peak velocity in patients with AF.

**Supplementary Information:**

The online version contains supplementary material available at 10.1186/s12872-023-03052-x.

## Introduction

The prevalence of heart failure (HF) is rising globally, affecting around 64.3 million people, with an age-standardised prevalence rate of 831 per 100 000 people [1, 2]. Echocardiography is usually the first imaging test performed to obtain information about left ventricular (LV) size and function, including LV ejection fraction (LVEF). Regardless of LVEF, which is preserved in almost half of the patients (heart failure with preserved ejection fraction, HFpEF) [3], an elevated LV filling pressure (LVFP) is almost always present in patients with HF to compensate and maintain the cardiac output [4]. Cardiac catheterisation remains the gold standard for LVFP estimation, although non-invasive methods of assessment have shown promising results [5, 6]. One of the diastolic indices used in haemodynamic measures for LVFP estimation is the mitral peak E-wave (peak velocity of transmitral blood flow in early LV diastole) and A-wave (peak mitral inflow velocity in late diastole due to atrial contraction) velocities. Mitral E-wave velocity represents the passive blood flow from the left atrium (LA) to LV, and mitral A-wave velocity reflects blood flow generated by active atrial contraction [7]. Any changes in LA contractility, LV compliance, or pressure gradient between LA and LV will result in an abnormal LVFP. Estimation of LVFP is crucial to diagnose and monitor the response to treatment in patients with HF.

Four-dimensional (4D) flow cardiac magnetic resonance imaging (CMR) is emerging as an important tool for LVFP estimation [5]. Compared to traditional Doppler echocardiography, 4D flow CMR provides a more precise and reproducible assessment of cardiac chamber function and volumes [8–11], especially in cases where echocardiographic windows can be challenging, yielding unreliable results [12].

This multicenter study sought to develop and evaluate the feasibility and accuracy of automated 4D flow CMR against Doppler echocardiography in estimating peak mitral inflow diastolic velocities using short-axis cine stack and examine its reproducibility.

## Methods

### Study cohort

This retrospective observational study included patients from the multicentre EurValve project (http://www.eurvalve.eu/) at Sheffield, UK and the Norfolk and Norwich University Hospital in Norwich, UK.

### Inclusion criteria

Adult patients with diagnosed HF who were stable as outpatients and had 4D flow CMR and standard Doppler echocardiography data were recruited for both sites. The exclusion criteria were limited to patients with severe aortic regurgitation and any MRI contraindications.

### Echocardiography

All echocardiograms were performed according to the British Society of Echocardiography guidelines for TTE examination [13]. For peak E-wave (early-filling) and peak A-wave (late-filling during atrial contraction) flow velocity measurements, pulsed-wave doppler TTE was used. Both measures were taken at the level of the tips of the mitral valve leaflets.

### Cardiac magnetic resonance

For Norwich data, CMR was done on a 3 Tesla Discovery 750w GE system (GE Healthcare, Milwaukee, WI, USA) equipped with an 8-channel cardiac coil. For Sheffield data, CMR was performed on a 3 Tesla Philips Healthcare Ingenia system equipped with a 28-channel coil and Philips dStream digital broadband MR architecture technology.

### CMR protocol

The CMR protocol used included a baseline survey and cines. Cine images were obtained during end-expiratory breath-hold with a balanced steady-state free precession (bSSFP) and single-slice breath-hold sequence. This protocol also included short axis cine and long axis cine SSFP in two-chamber, three-chamber and four-chamber views. For each pulse sequence, images with aliasing artefacts were repeated until any artefact was removed or excluded, and only the highest-quality images were used for analysis.

### 4D flow CMR acquisition

The initial VENC setting was 150–200 cm/sec for all 4D flow CMR acquisition cases. Generic MRI parameters were similar on both Philips and GE systems. The field of view was planned to cover the whole heart, aortic valve and ascending aorta. The Philips system used echo-planar imaging (EPI) acceleration factor of 5 with no respiratory gating [10, 14]. On the GE system, HYPERKAT acceleration with a factor of 2 was used [15]. Other standard scan parameters were: field-of-view = 340 mm × 340 mm, acquired voxel size = 3 × 3 × 3 mm3, reconstructed voxel size = 1.5 × 1.5 × 1.5 mm3, echo time (TE) = 3.5 ms, repetition time (TR) = 10 ms, flip angle = 10°, and 30 cardiac phases.

### 4D flow CMR image analysis

**Fig. 1:**
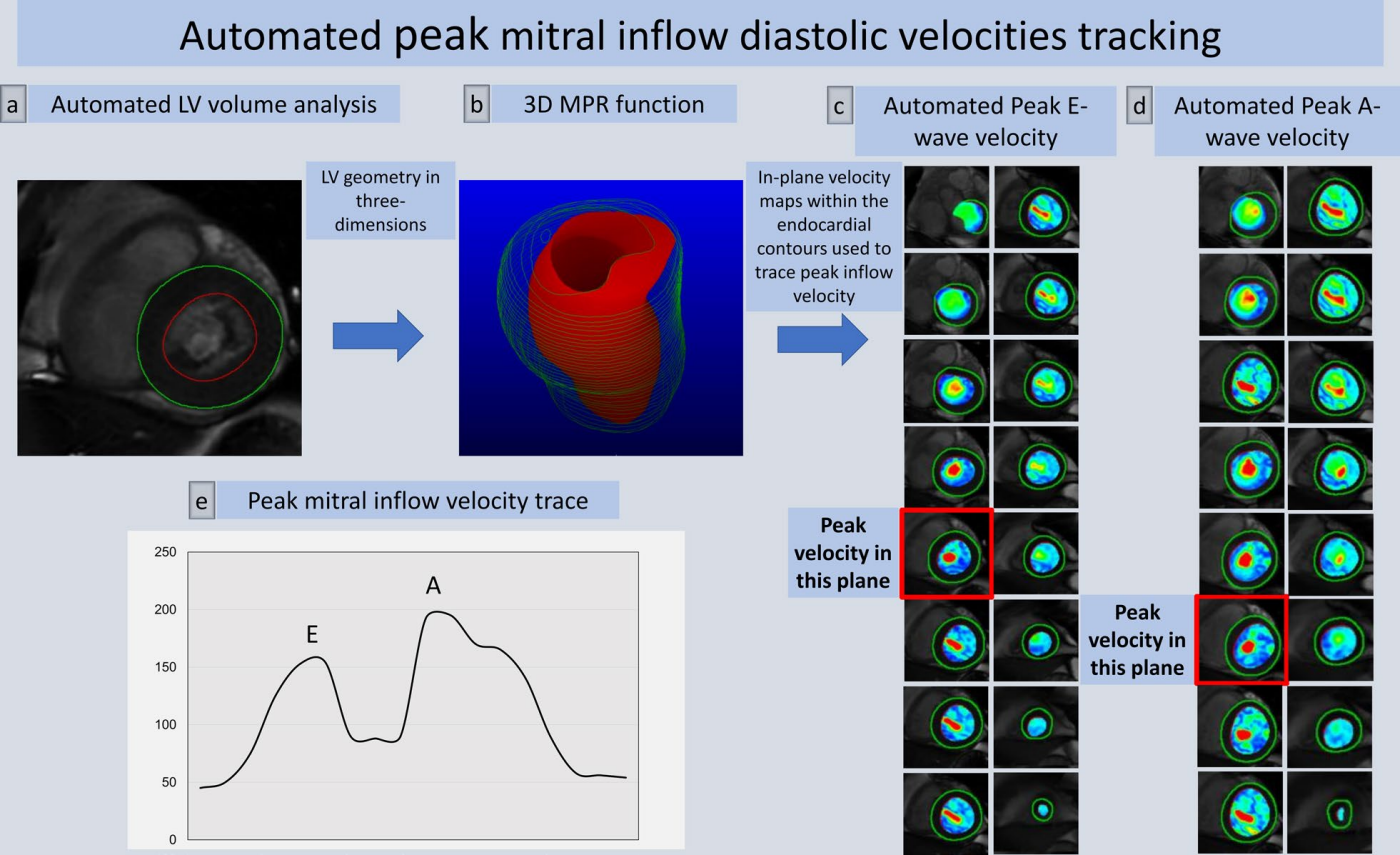
Peak mitral inflow velocity tracking on MASS using 4D flow CMR. The software automatically computes in-plane velocity maps within the contoured area for the complete cardiac cycle. Automated analysis of the LV volumes from short-axis cine stack of images using standard methods of endocardial and epicardial contours (**a**). Three-dimensional multi-planar reformatted plane showing the LV geometry in 3D (**b**). Short-axis cine stack view and three-directional blood flow images showing mitral inflow as color-coded velocity maps on 4D flow CMR during diastole (**c**, **d**). Peak mitral inflow velocity trace demonstrating peak E-wave and peak A-wave velocities (**e**)

Transvalvular 4D flow analysis through the mitral valve and peak velocity quantification were post-processed with the in-house developed MASS research software (MASS; Version 2019-EXP, Leiden University Medical Center, Leiden, The Netherlands). The protocol we used for peak mitral inflow velocity assessment is described in Fig. 1. LV volumes were segmented in the stack of short-axis cine images. Prior to quantification, any spatial misalignment with cine superimposition was manually corrected throughout the cardiac cycle. Peak E-wave and peak A-wave mitral inflow velocities were identified during diastole in the 4D flow data set and were recorded.

The steps taken to identify the peak mitral inflow diastolic velocities were as follows:

1. A multi-planar reformatted (MPR) stack of short-axis cine images with 60 slices, 0 mm thickness, 2–3 mm spacing and 90-degree angle was generated using the initial centerline method.
2. Automated analysis of the LV volumes from cine images using standard methods of endocardial and epicardial contours in all phases was performed [16].
3. The software automatically computed in-plane velocity maps within the contoured area of mitral inflow and identified the peak velocity with the streamlines for the complete cardiac cycle.
4. Finally, peak E-wave and peak A-wave velocities were recorded from the maximum velocity graph generated by the software. Only the peak E-wave velocity was recorded for atrial fibrillation (AF) patients.

More details of these steps are described in Additional file 1: Figure S1.

### Intra- and inter-observer variability

For intraobserver analysis, H.A. (2-years CMR experience) repeated the analysis for 30 cases after three months. For interobserver analysis, a second investigator R.L. (3-months CMR training), performed the analysis in 20 random cases.

### Statistical analysis

Data analyses were performed using SPSS (version 28.0, IBM, Chicago, Illinois, USA) and confirmed in MedCalc (MedCalc Software, Ostend, Belgium version 20.011). Continuous variables were expressed as median ± interquartile range (IQR). Normality and lognormality testing was performed for all data using the Shapiro–Wilk test before the analysis. The Wilcoxon test was performed to compare the difference between the median mitral inflow velocities measured by Doppler TTE and 4D flow CMR. Correlations between the two imaging modalities were evaluated using Spearman’s coefficient of rank correlation (r) and reported with 95% confidence intervals (95% CI). Bland–Altman plots were constructed to evaluate the agreement between Doppler TTE and 4D flow CMR. For intra-and inter-observer variability, reproducibility analyses were performed and reported by the coefficient of variation (CoV) using the logarithmic method. The significance threshold was set at *P* < 0.05.

## Results

### Patient characteristics

Forty-eight patients were included in this study (32 from the EurValve project and 16 from Norwich). Patient demographics and clinical characteristics are summarised in Table 1. The median age was 69 years (IQR 63 to 76 years), and 54% were males. 83% were in sinus rhythm, 35% were hypertensive, and 20% were diabetic. Nearly half of the patients (42%) were smokers. One-third were in New York Heart Association (NYHA) class I (35%) and II (31%); 8% were in NYHA class III, and 4% had a previous history of myocardial infarction. Patients were on a range of long-term medications, including beta-blockers and diuretics (45%), angiotensin-converting enzyme inhibitors (19%), angiotensin receptor antagonists (8%), and calcium channel blockers (6%).

**Table 1:** Demographic variables of the 48 patients included in this study

Characteristics	Median ± IQR or N (%)
Age (years)	69 ± 12
BSA (m^2^)	1.8 ± 0.3
Gender (Male)	26 (54%)
Co-morbidities	
Atrial fibrillation	8 (17%)
Diabetes mellitus	10 (20%)
Hypertension	17 (35%)
Previous myocardial infarction	2 (4%)
Current or Ex-smoker	20 (42%)
NYHA classification	
NYHA I	17 (35%)
NYHA II	15 (31%)
NYHA III	4 (8%)
Medications	
Beta-blockers	12 (25%)
Loop diuretics	5 (10%)
Other diuretics	5 (10%)
Calcium-channel antagonists	3 (6%)
Angiotensin-receptor antagonists	4 (8%)
Angiotensin-converting enzyme inhibitors	9 (19%)

### Correlation

**Fig. 2:**
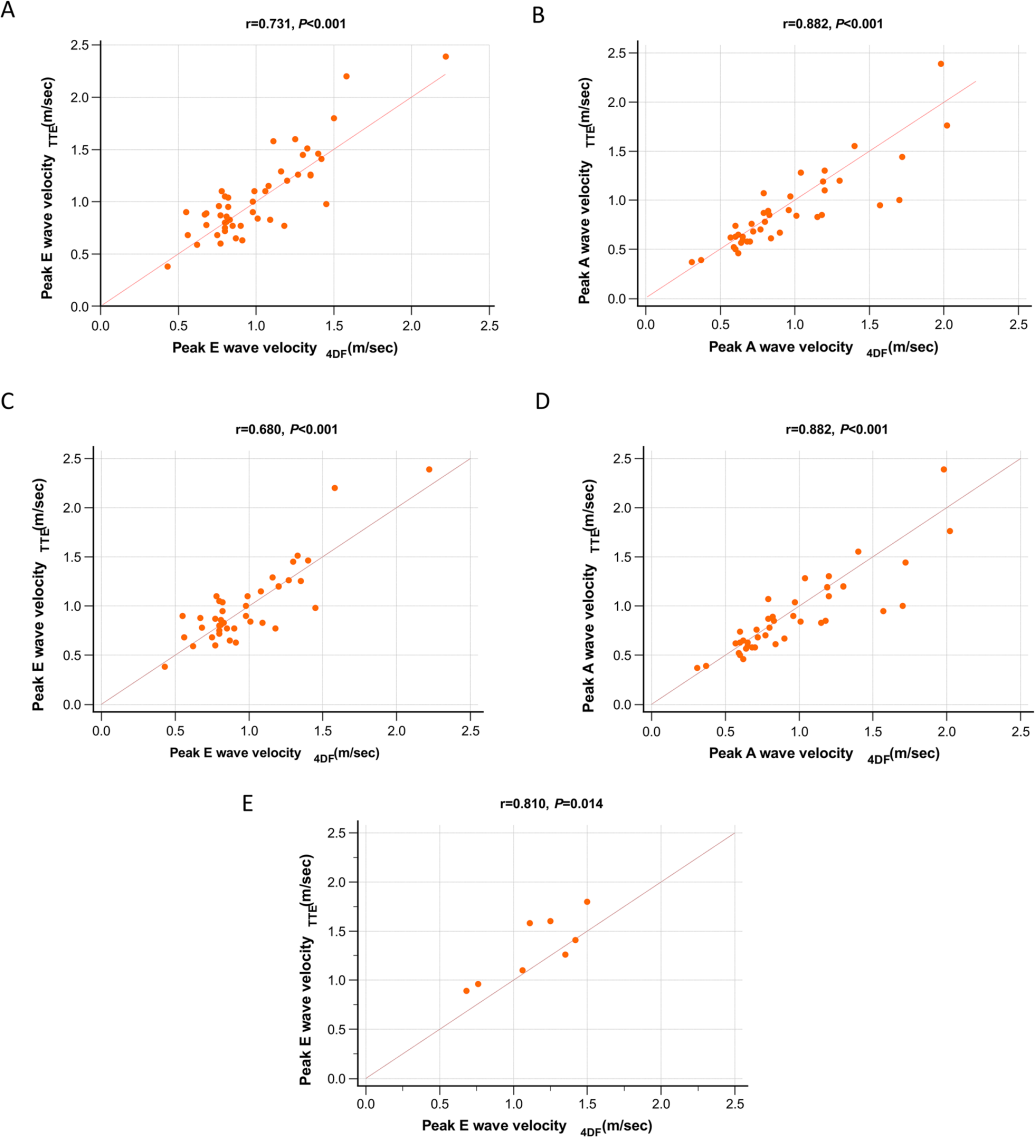
Scatter plots demonstrating correlations between Doppler TTE peak mitral inflow velocity readings and 4D flow CMR peak mitral inflow velocity measurements. **A***, ***B** All patients. **C***, ***D** Sinus rhythm-only patients. **E** AF-only patients

Automated 4D flow CMR strongly correlated with Doppler TTE when measuring peak E velocity in all patients (r = 0.73, 95% CI 0.56–0.84, *P* < 0.001) and peak A mitral inflow velocity (r = 0.88, 95% CI 0.79–0.94, *P* < 0.001). Moreover, there was a significant correlation between automated 4D flow CMR and TTE for peak-E wave velocity in the sinus rhythm-only group (r = 0.68, 95% CI 0.47–0.82, *P* < 0.001) and AF-only group (r = 0.81, 95% CI 0.24–0.96, *P* = 0.014). (Fig. 2).

### Agreement

No significant differences were observed between Doppler TTE and 4D flow CMR when measuring peak mitral E-wave velocity in all cases (0.94 ± 0.48 m/s vs 0.90 ± 0.43 m/s, *P* = 0.13) and peak mitral A-wave velocity (0.81 ± 0.44 m/s vs 0.81 ± 0.53 m/s, *P* = 0.09), respectively. Moreover, the agreement remained the same when measuring peak E-wave velocity in sinus rhythm-only group (0.88 ± 0.35 m/s vs 0.86 ± 0.38 m/s, *P* = 0.54) and in AF-only group of patients (1.33 ± 0.56 m/s vs 1.18 ± 0.47 m/s, *P* = 0.06) (Table 2).

**Table 2:** Comparison between median mitral inflow peak diastolic velocity measurements by transthoracic echocardiography and automated 4D flow CMR using Wilcoxon test

	Peak E-wave velocity (m/s)	Peak A-wave velocity (m/s)
TTE		
All	0.94 ± 0.48	0.81 ± 0.44
Sinus rhythm	0.88 ± 0.35	0.81 ± 0.44
AF	1.33 ± 0.56	–
4D flow CMR		
All	0.90 ± 0.43	0.81 ± 0.53
Sinus rhythm	0.86 ± 0.38	0.81 ± 0.53
AF	1.18 ± 0.47	–
*P*value		
All	0.13	0.09
Sinus rhythm	0.54	0.09
AF	0.06	–

Median ± IQR

**Fig. 3:**
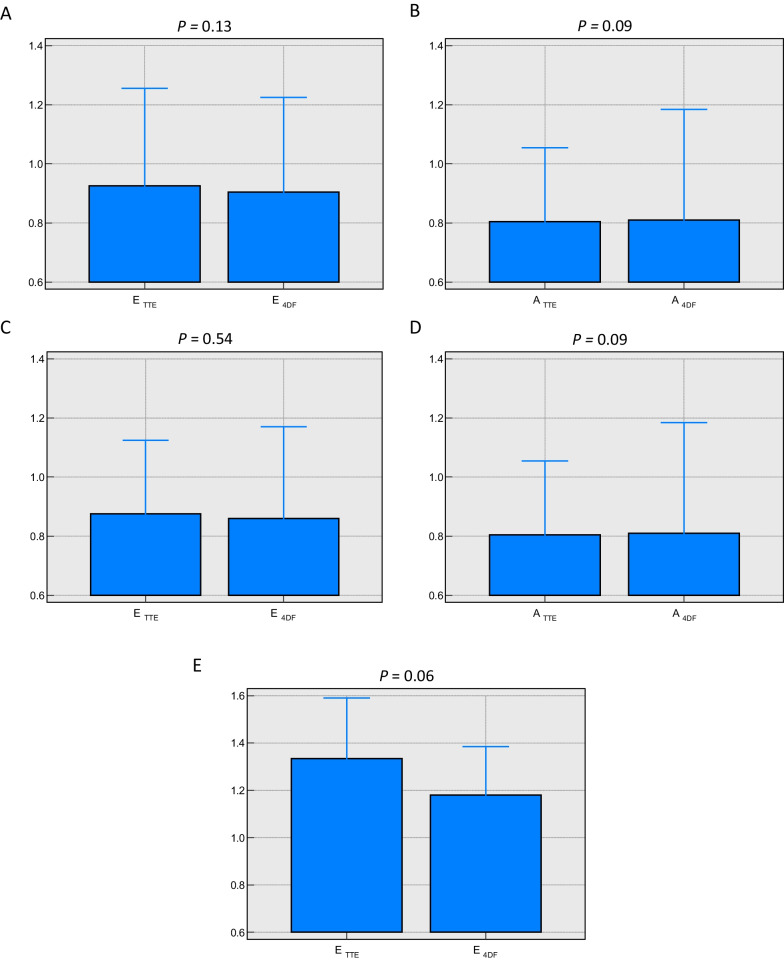
Bar Charts illustrating the comparison of median mitral inflow velocities between Doppler TTE and 4D flow CMR, In all patients group (**A**, **B**), sinus rhythm-only group (**C**, **D**) and AF-only group (**E**)

**Fig. 4:**
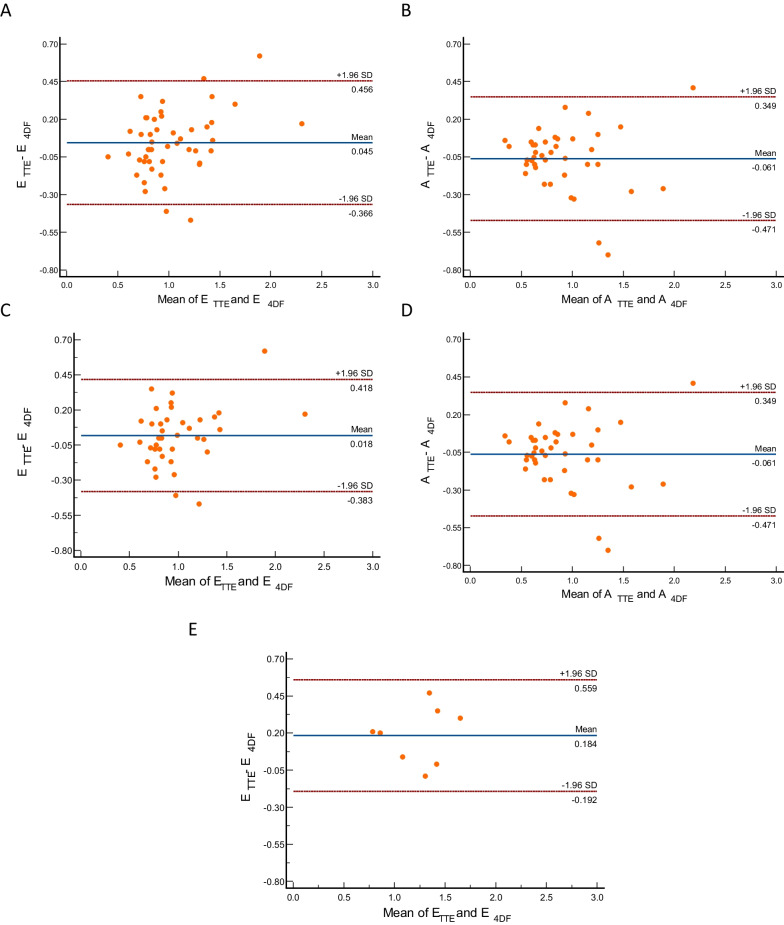
Bland–Altman plots demonstrating the degree of agreement between Doppler TTE and 4D flow CMR to measure the peak E wave and A wave mitral inflow velocity parameters. **A**, **B** All patients. **C**, **D** Sinus rhythm-only group. **E** AF-only group of patients

On Bland–Altman analyses, the mean bias in peak E-wave velocity between Doppler TTE and automated 4D flow CMR in all patients was 0.05 m/s (95% CI − 0.37 to 0.46 m/s, *P* = 0.14), sinus rhythm-only patients 0.02 m/s (95% CI − 0.04 to 0.08 m/s, *P* = 0.6) and AF-only patients 0.18 m/s (95% CI 0.02 to 0.34 m/s, *P* = 0.03). For peak A-wave velocity, the mean bias between Doppler TTE and automated 4D flow CMR in all cases was − 0.06 m/s (95% CI − 0.47 to 0.35 m/sec, *P* = 0.07). Bland–Altman plots and bar charts illustrating the agreement between Doppler TTE and 4D flow CMR methods in both measured parameters of peak diastolic mitral inflow velocity in the three groups are shown in Fig. 3 and Fig. 4.

### Intra- and inter-observer variability

Measures of intra- and inter-observer variability are provided in Table 3. On a subgroup of 30 patients selected randomly from both centres, the intra-observer CoV for E-wave and A-wave velocities were 2.2% and 3.5%, respectively. On a subgroup of 20 patients, inter-observer CoV for peak E-wave and peak A-wave velocities were 4.9% and 4.3%, respectively. The 4D flow CMR post-processing times took about 12–15 min.

**Table 3:** Intraobserver and interobserver repeatability analysis for 4D flow CMR

	Intraobserver (CoV*) (%)	Interobserver (CoV*) (%)
MV peak E-wave velocity	2.2	4.9
MV peak A-wave velocity	3.5	4.3

Coefficient of variation*

## Discussion

Our study sought to investigate the agreement between Doppler TTE and automated 4D flow CMR for deriving peak mitral inflow diastolic velocities using the short-axis cine stack. We have demonstrated that automated 4D flow CMR yields similar peak E-wave and peak A-wave velocities as standard Doppler pulse-wave TTE. Furthermore, automated 4D flow CMR displayed excellent intraobserver and interobserver repeatability for peak mitral inflow velocities.

HFpEF is associated with poor prognosis [17–19] and can be challenging to diagnose and manage [20]. Peak mitral inflow diastolic velocities are vital for estimating diastolic function and LVFP measurements and have substantial prognostication value when done accurately [7]. Although echocardiography has been a cornerstone in diastolic function assessment for decades, a range of limitations (including operator dependency and narrow acoustic windows) make it an imprecise technique for clinical application [21]. With new medications like SGLT2 inhibitors likely to be beneficial in HFpEF, it would be important to identify this early on [22]. CMR is an advanced imaging tool and is the gold standard for non-invasive assessment of cardiac chamber volumes, function and structure. CMR offers an unrestricted field of view and excellent spatial resolution compared to TTE for an improved assessment of structural abnormalities. It can also assist assessment of cardiac function in patients with structural abnormalities, offering a complementary role to echocardiography [23]. 4D flow CMR classifies intracardiac flow into direct flow (blood entering and exiting chambers), retained inflow (blood entering but not exiting), delayed ejection flow (blood exiting on the next heartbeat) and residual volume (blood residing for more than one cycle) [24]. Flow differences missed by echocardiography are easily recognisable by 4D flow CMR. Automated 4D flow offers feasible and rapid operator-independent flow quantification, with excellent reproducibility for clinical use [25–27].

Previous studies have investigated the performance of 4D flow CMR for assessing peak diastolic velocities in cases where TTE is suboptimal. Njoku et al*.* [12] demonstrated that 4D flow CMR could easily quantify peak mitral inflow velocities in aortic regurgitation, where echocardiography falls short. They used novel 3D peak velocity tracing by 4D flow CMR and were able to track blood flow at the mitral annulus [12]. Other studies compared different 4D flow CMR methods that have been user-dependent and time-consuming. Kamphuis et al*.* presented a single automated method demonstrating rapid analysis with strong intra and interobserver variability to overcome those limitations [25]. Other studies have investigated the accuracy and reproducibility of peak diastolic velocities using 4D flow CMR against Doppler echocardiography [28–31]. Our study showed a strong correlation and excellent intraobserver and interobserver repeatability with no significant differences in peak mitral inflow diastolic velocities. The results of our study confirm the findings of our previous study using the four-chamber cine stack and further supports the adoption of 4D flow CMR for routine assessment of LV diastolic function [31]. Previous literature has also demonstrated that CMR offers superior results to echocardiography when measuring LVFP compared with invasive methods in patients with suspected HF [5]. Additionally, the prognostic value of peak E wave velocity in patients with asymptomatic mitral regurgitation (MR) and preserved LV function has been demonstrated in a study by Okamoto et al*.* [32] using Doppler echocardiography, which needs to be investigated using 4D flow CMR.

### Limitations

Firstly, manual adjustments of spatial misalignment and aliasing artefacts are current limitations of CMR and may introduce user-dependent variation. Quality improvements with CMR acquisition may eliminate this limitation and further improve reproducibility. Secondly, echocardiography and CMR were not performed simultaneously, which may introduce haemodynamic variation. The average time difference across all cases was 19 months which could explain the broader limits of agreement shown in our Bland–Altman analysis. However, despite this, we have demonstrated a significant correlation which could have been improved if the CMR and echo were done on the same day. Thirdly, patients with severe aortic regurgitation were excluded from our study as our technique tends to overestimate the peak flow velocities in these patients. We hope that in the future, we will be able to exclude the reverse flow from the LV outflow tract into our model. Finally, contrary to echocardiography, 4D flow CMR is yet to become a standard component in clinical practice because of the lengthy post-processing protocol and exorbitant analysis software. More work must be done to develop a more user-friendly, accessible analysis software to support its adoption in routine practice.

## Conclusion

Automated dynamic peak mitral inflow diastolic velocity tracing using automated 4D flow CMR is comparable to Doppler echocardiography and has excellent repeatability for clinical use. We propose that the clinical utility of 4D flow CMR for routine assessment might be viewed as equivalent to that of Doppler echocardiography. Future prospective studies are needed to investigate the diagnostic and prognostic yield of automated 4D flow CMR in patients with HFpEF and patients with MR.

## Supplementary Information

**Additional file 1: Supplementary Figure 1.** Steps taken to identify the peak mitral inflow diastolic velocities.

## Data Availability

The datasets generated and analysed during the current study are not publicly available. Access to the raw images of patients is not permitted since specialised post-processing imaging-based solutions can identify the study patients in the future. Data are available from the corresponding author upon reasonable request.

## Abbreviations

4D flow: Four-dimensional flow
AF: Atrial fibrilation
CMR: Cardiac magnetic resonance imaging
CoV: Coefficient of Variation
HF: Heart failure
LA: Left atrium
LVEF: Left ventricular ejection fraction
LVFP: Left ventricular filling pressure
MV: Mitral valve
NYHA: New York Heart Association
TTE: Transthoracic echocardiography
