# Supplementary figures and images for: Automated 4D flow cardiac MRI pipeline to derive peak mitral inflow diastolic velocities using short-axis cine stack: two centre validation study against echocardiographic pulse-wave doppler
Source: BMC Cardiovasc Disord. 2023 Jan 16;23:24. doi: 10.1186/s12872-023-03052-x (PMC9843884; doi:10.1186/s12872-023-03052-x)

**Supplementary Figure 1** - Steps taken to identify the peak mitral inflow diastolic velocities.


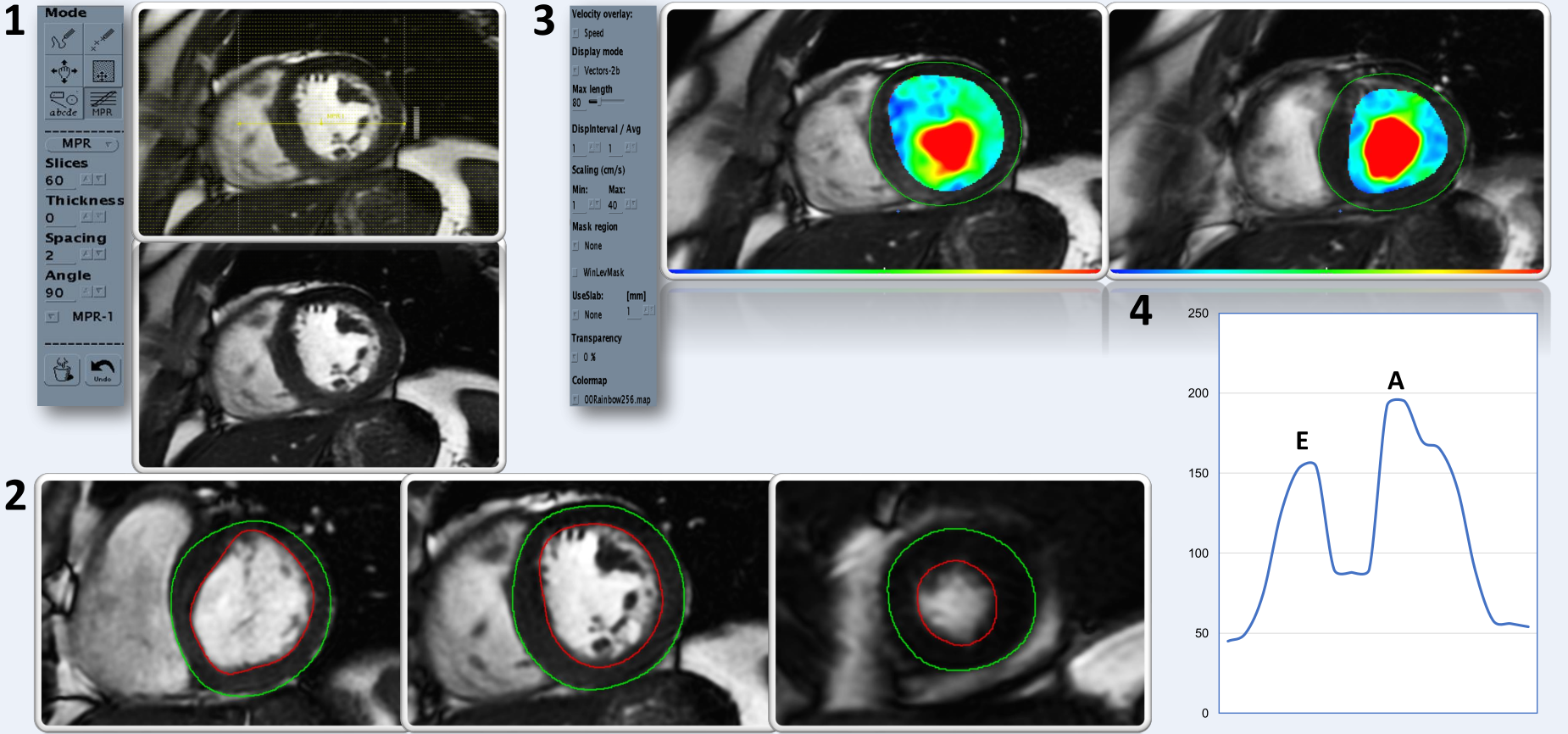

Supplement: Supplementary file 1 — Additional file 1: Supplementary Figure 1. Steps taken to identify the peak mitral inflow diastolic velocities. [file 12872_2023_3052_MOESM1_ESM.docx]
